# Supplementary figures and images for: Genetic diversity and demography of Bufo japonicus and B. torrenticola (Amphibia: Anura: Bufonidae) influenced by the Quaternary climate
Source: PeerJ. 2022 Jun 8;10:e13452. doi: 10.7717/peerj.13452 (PMC9188313; doi:10.7717/peerj.13452)

(a)

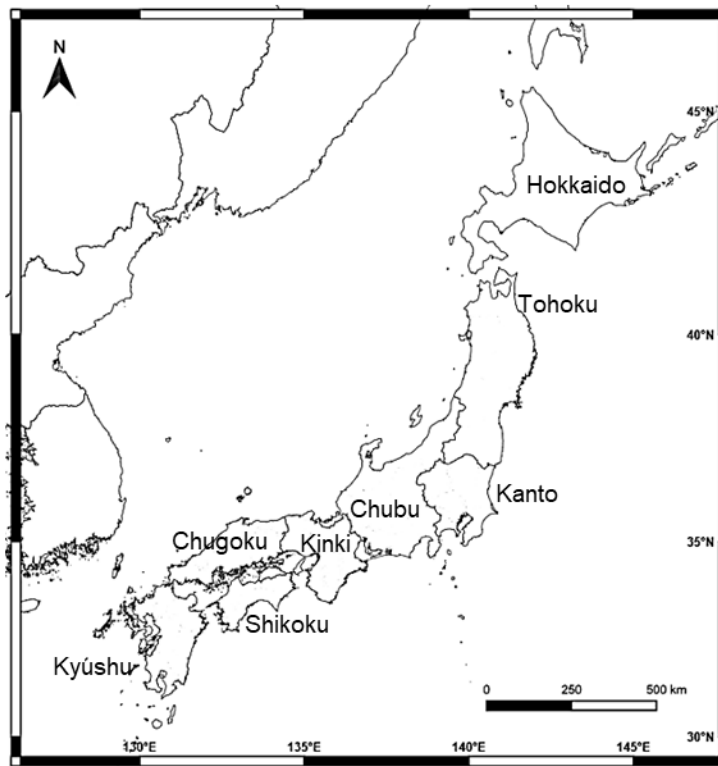

(b)

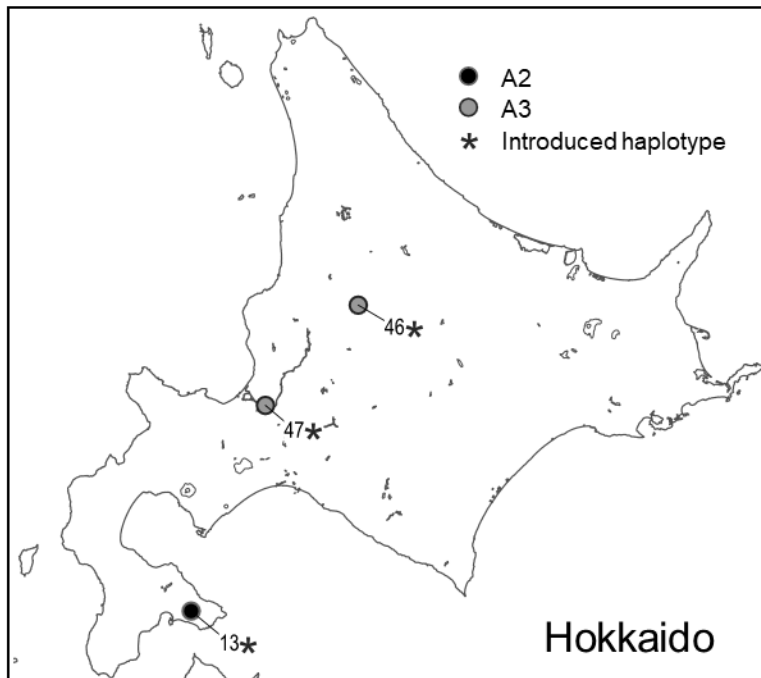

(c)

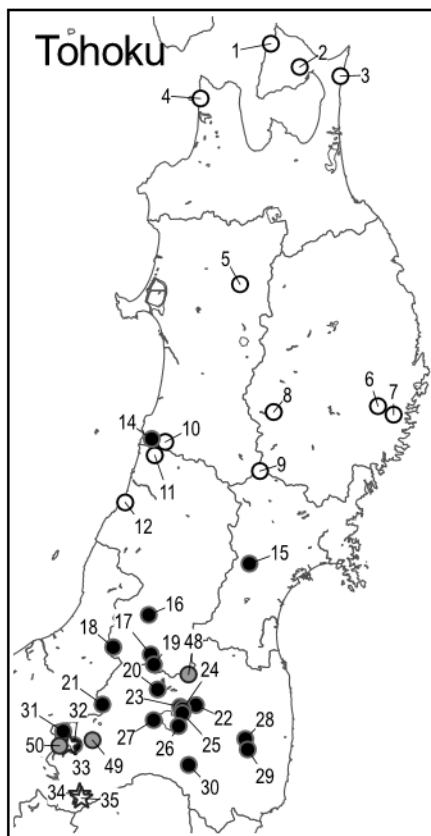

- A1
- A2
- A3
- ▼ B1
- ☆ A2 and A3 (locality 33–35)
- ☆ A3 and B1 (locality 67)
- \* Introduced haplotype

(d)

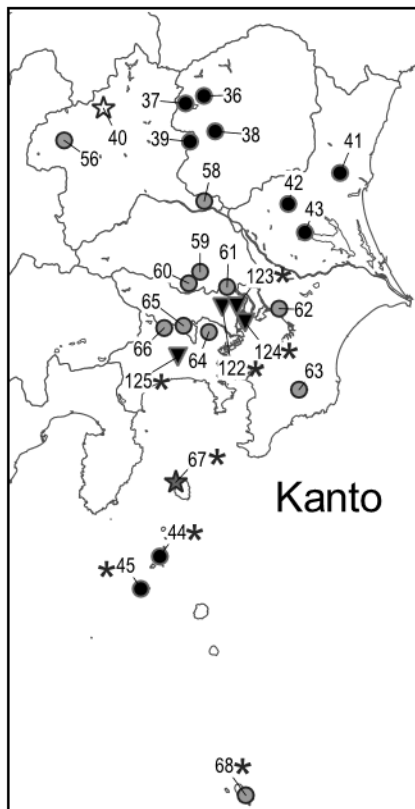

(e)

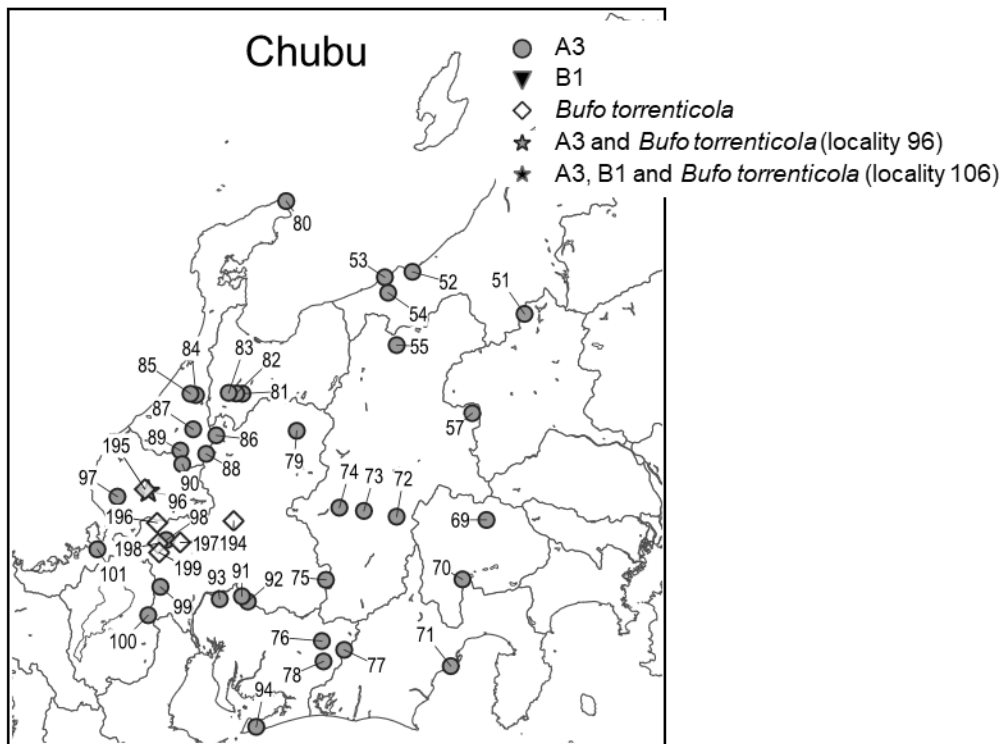

(f)

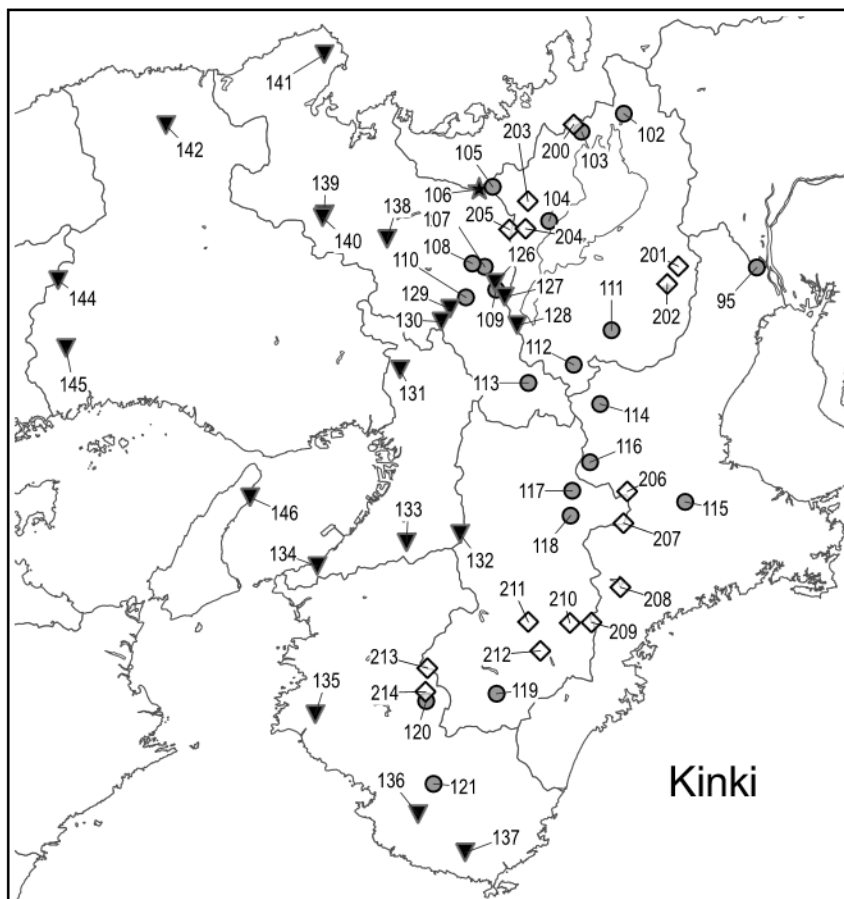

(g)

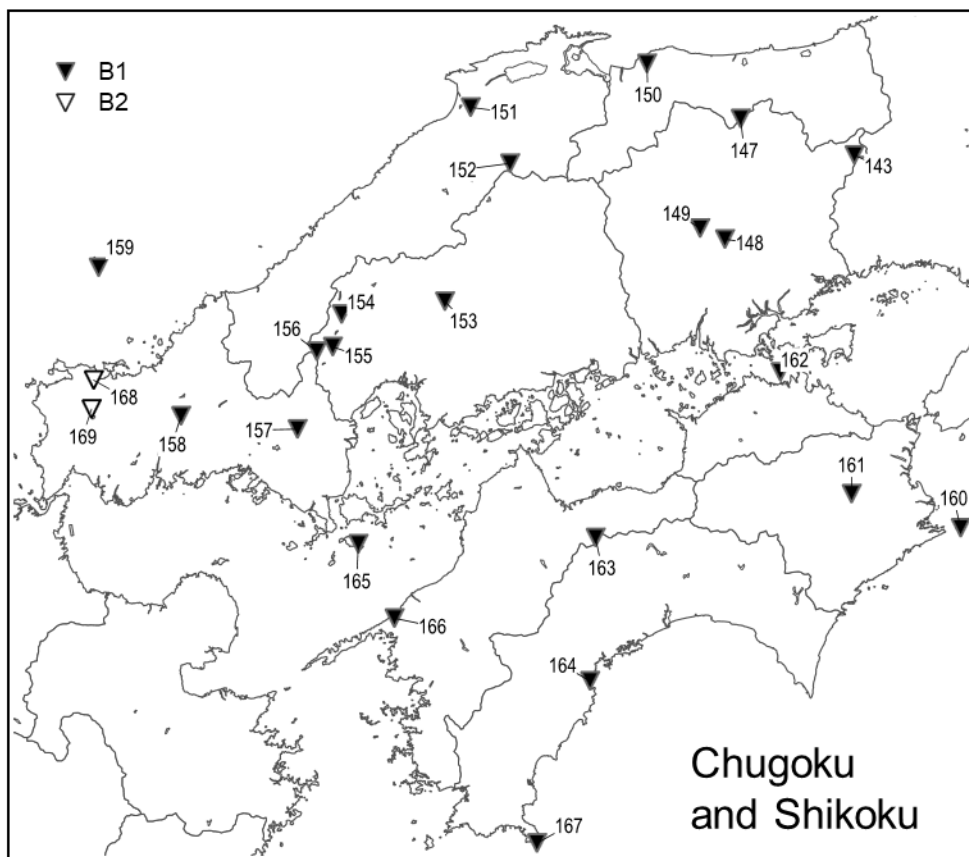

(h)

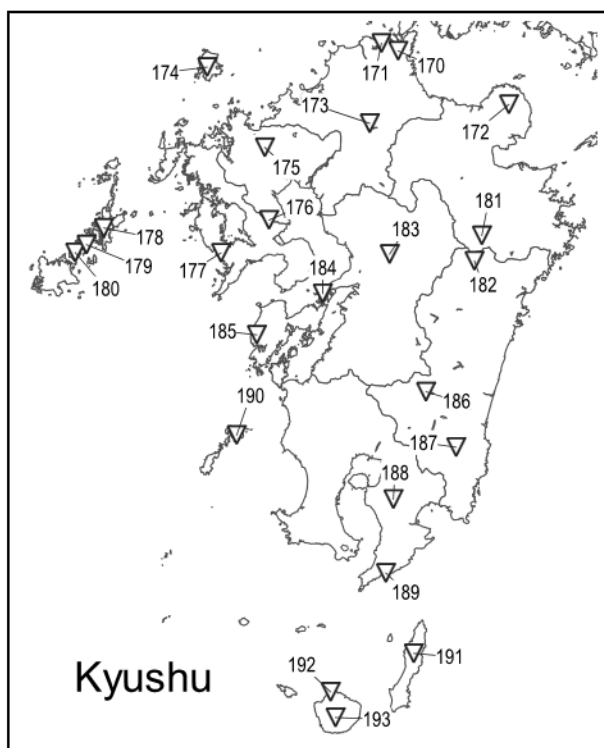

Supplement: Supplemental Information 1 — Clade A1, white circles; clade A2, black circles; clade A3, gray circle; clade B1, black inverted triangle; clade B2, white inverted triangle; B. torrenticola, white diamond. Stars indicate localities with the sympatry of several clades of B. japonicus or B. torrenticola. Asterisks indicate localities with identified introduced haplotypes. Regarding locality information, see Table S1. Maps were created by QGIS 3.16 (https://qgis.org). [file peerj-10-13452-s001.pdf]

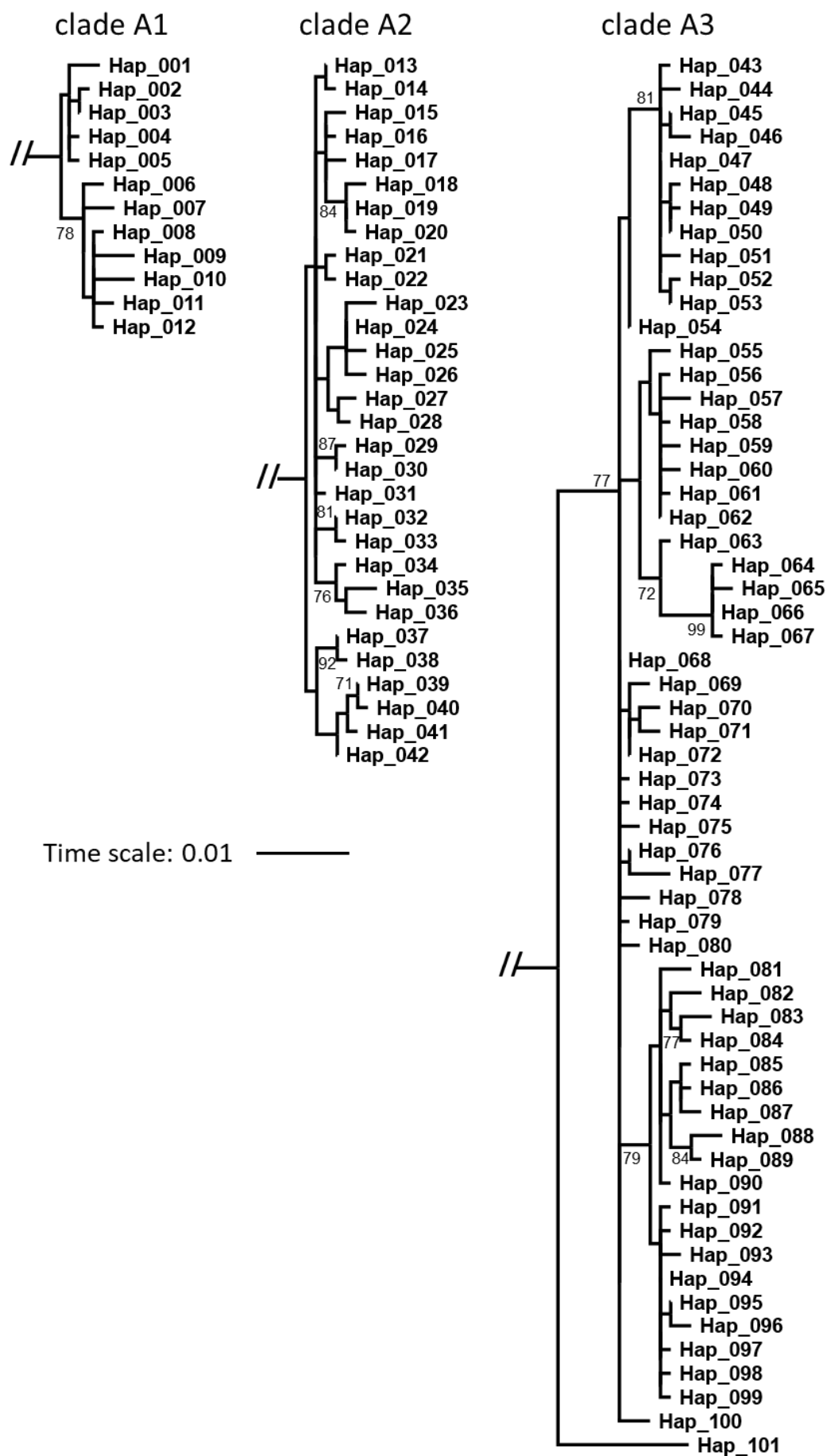

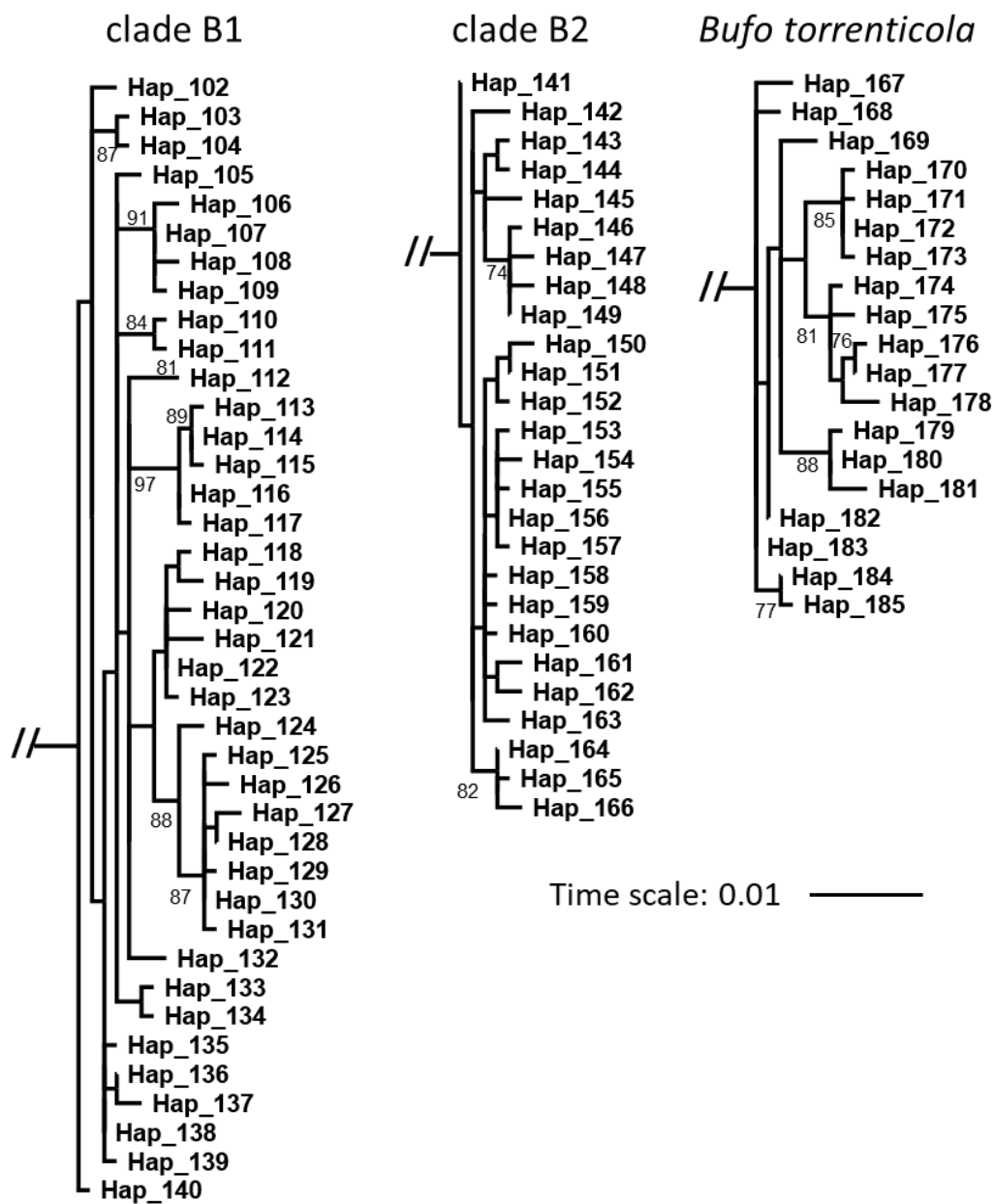

Supplement: Supplemental Information 2 — Topologies and branch lengths are based on the Maximum Likelihood (ML) tree. Nodal Numbers represent ML bootstrap supports (>70%). The scale bar indicates substitutions per site. Regarding haplotype numbers, refer to Table S1. Trees were visualized by FigTree v.1.4.4 (tree.bio.ed.ac.uk/software/figtree/). [file peerj-10-13452-s002.pdf]

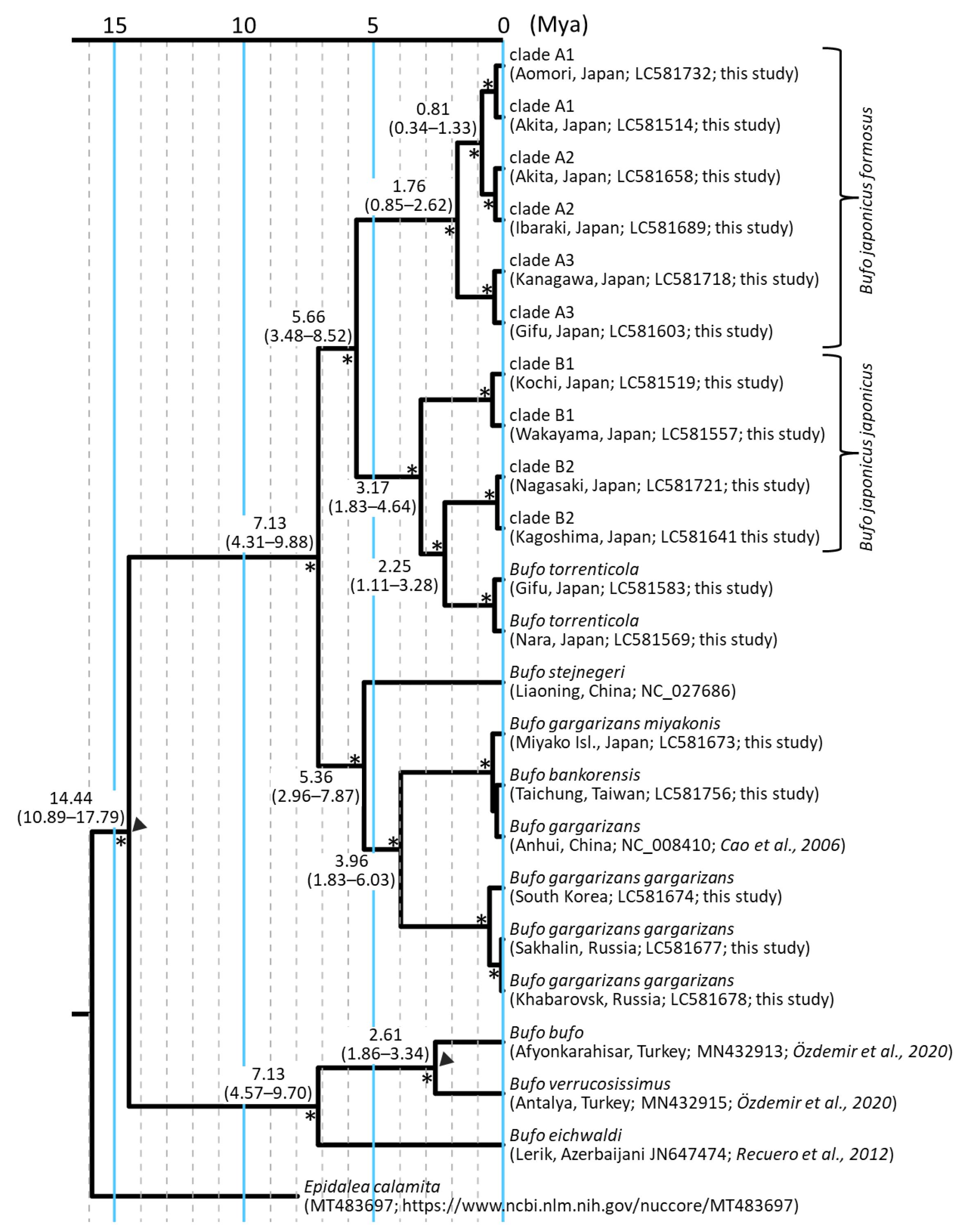

Supplement: Supplemental Information 3 — Divergence times were estimated with two calibrations. Estimated divergence times and 95% HPD (Mya) are shown around the main nodes. Nodes are placed based on mean node ages. Asterisks represent nodes with posterior probabilities with values over 0.95. Two triangles indicate the calibration points used to obtain the time tree. The scale bar indicates substitutions per site. The tree was visualized by iTOL v6 (Letunic & Bork, 2021). [file peerj-10-13452-s003.png]

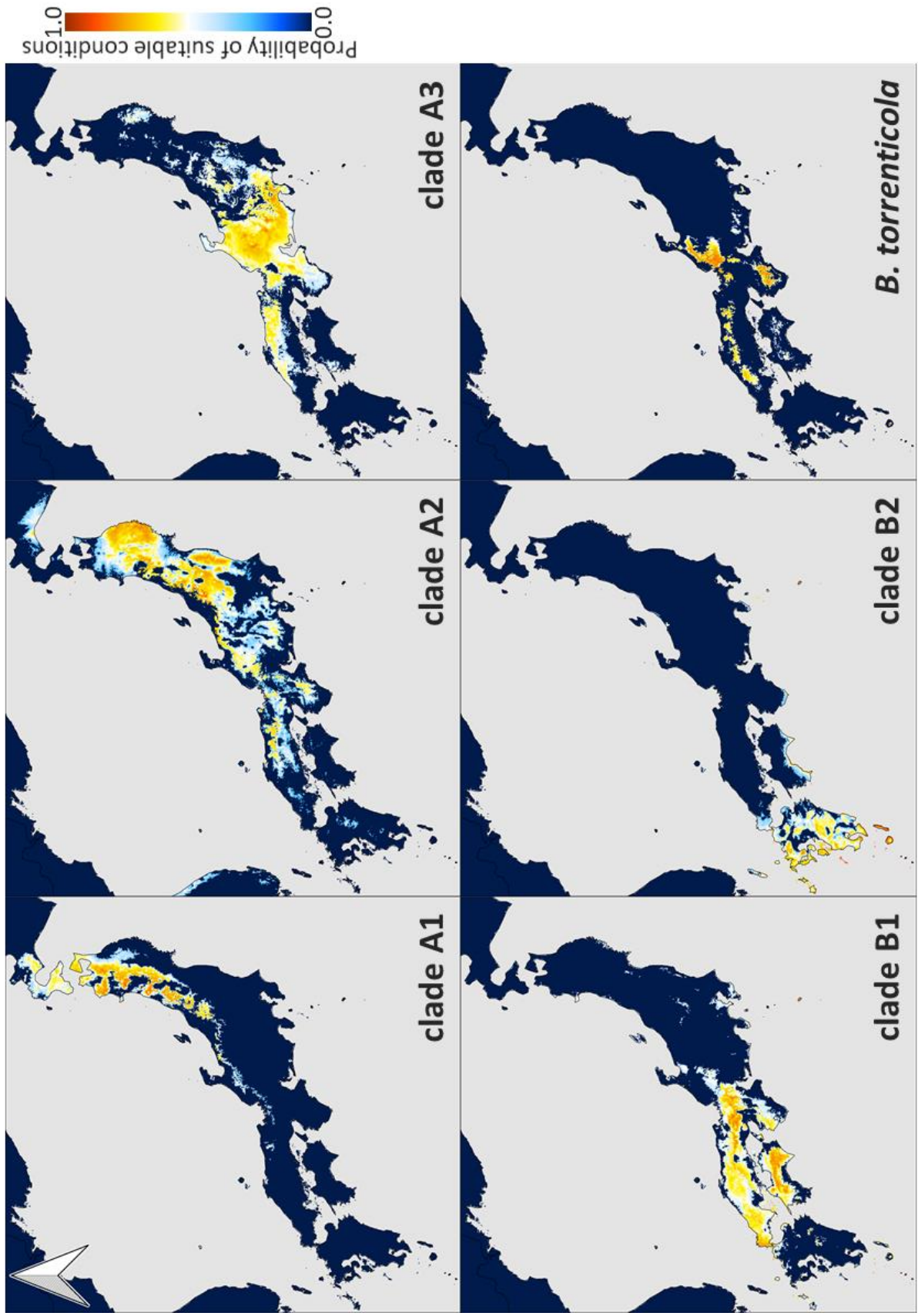

Supplement: Supplemental Information 4 — Warmer colors indicate higher probabilities of occurrence. Navy blue zones, land areas; grey zones, oceanic areas. Maps were created using R package map data version 2.3.0 (Becker, Wilks & Brownrigg, 2018). [file peerj-10-13452-s004.pdf]
